# How does supervisor support shape PhD students’ learning experience? Evidence from the Nature Careers 2025 Global PhD Survey

**DOI:** 10.1371/journal.pone.0357413

**Published:** 2026-09-01

**Authors:** Nuo Ya Li, Hanjie Zhang, Junqi Wang, Mingyu Ye

**Affiliations:** 1 Department of Basic Education, Nanjing Vocational Institute of Railway Technology, Nanjing, Jiangsu, China; 2 Institute of Education, Nanjing University, Nanjing, Jiangsu, China; 3 School of Business, Xinjiang Normal University, Urumqi, Xinjiang, China; 4 Faculty of Education, The University of Hong Kong, Hong Kong, China; King Abdulaziz University Faculty of Medicine, SAUDI ARABIA

## Abstract

Supervisory support is central to doctoral students’ learning experiences, yet the relative importance of contact frequency and support quality remains unclear. Using publicly available data from 3,785 PhD students in 107 countries in the Nature Careers 2025 Global PhD Survey, this study examines how supervisory contact frequency, relationship quality, research guidance, career guidance, and career support are associated with overall PhD satisfaction. Ordinary least squares regression provides the baseline estimates, supplemented by ordered logit and binary logit models and alternative-outcome and extended-control checks. Contact frequency is positively associated with satisfaction in descriptive and reduced models, but its independent association becomes weak and unstable after support-quality dimensions are introduced. In contrast, supervisory relationship quality, research guidance, career guidance, and career support remain positively and consistently associated with satisfaction across model specifications. These dimensions are also associated with whether students perceive their doctoral experience as meeting their initial expectations. The findings indicate that the quality and developmental content of supervisory interaction matter more than contact time alone. By disaggregating supervisory support, this study contributes global evidence to doctoral education research and informs institutional efforts to strengthen supervision quality, feedback, and career development support.

## Introduction

Doctoral education occupies the highest level of the higher education system. Its quality is closely related not only to the supply of highly trained talent for universities and research institutions, but also to knowledge production, academic innovation, and the long-term development of professional labour markets. In recent years, PhD students’ learning experience has become an increasingly important topic in higher education research. Compared with undergraduate and taught postgraduate education, doctoral learning is more prolonged, individualized, and uncertain. PhD students are expected to complete research training, dissertation writing, academic publishing, career decision-making, and identity transition over an extended period. Their evaluation of the doctoral experience is therefore shaped not only by research outputs or coursework, but also by the support relationships embedded in the training process.

Within doctoral education, supervisors constitute one of the most important sources of institutional support. Supervisors influence PhD students’ research directions, methodological choices, and dissertation progress. They also shape students’ overall perceptions of the doctoral experience through feedback practices, communication quality, career advice, and access to development opportunities. Existing English-language studies have widely shown that supervisor support is closely associated with PhD students’ satisfaction, research engagement, mental health, and academic persistence [1–3]. This body of research has moved doctoral education studies beyond a narrow focus on completion rates and research outputs toward a stronger concern with students’ lived experiences during doctoral training.

Nevertheless, existing discussions of supervisor support still require further refinement. On the one hand, many studies treat supervisor support as an overall construct, which may obscure differences among its internal dimensions. The support PhD students receive from supervisors is not limited to research guidance; it also includes relational support, career guidance, and encouragement for diverse career pathways. On the other hand, the distinction between supervisory contact frequency and the quality of supervisor support has not been sufficiently clarified. In doctoral training practice, increasing the amount of supervisor–student contact is often regarded as a direct way to improve supervision quality. Yet contact frequency does not necessarily imply effective support. Frequent contact may provide timely feedback, but it may also involve more intensive administrative communication, progress pressure, or formalized interaction. What may matter more for PhD students’ learning experience is not the amount of contact itself, but whether the interaction contains clear, trustworthy, and development-oriented support.

This issue is particularly relevant in the current context of doctoral education. As the career destinations of PhD graduates become increasingly diverse, doctoral training no longer serves only traditional academic career pathways. PhD students need not only guidance on dissertation writing and research methods, but also opportunities to discuss academic, non-academic, and cross-sector career development. Whether supervisors are willing to discuss career issues openly, accept non-academic career choices, provide relevant advice, and encourage students to participate in career development activities has become an important component of how PhD students evaluate their training experience. If supervisor support continues to be understood merely as academic supervision, it becomes difficult to explain the complexity of contemporary doctoral learning experiences.

Against this background, this study conducts a secondary analysis of the publicly available anonymized data from the Nature Careers 2025 Global PhD Survey [4]. Drawing on responses from 3,785 PhD students across 107 countries, it examines how supervisor support is associated with PhD students’ learning experience. Overall PhD satisfaction is used as the main outcome variable. Supervisor support is divided into two dimensions: supervisory contact frequency and the quality of supervisor support. The latter is further specified as supervisory relationship quality, research guidance, career guidance, and supervisor career support. Methodologically, ordinary least squares regression is used as the baseline model, supplemented by ordered logit models, binary logit models, alternative outcome models, and models with extended control variables for robustness checks.

The central purpose of this study is not simply to verify whether supervisor support matters, but to compare the relative explanatory roles of supervisory contact frequency and support quality. The empirical results show that supervisory contact frequency is significantly associated with overall PhD satisfaction in simpler models. However, after supervisory relationship quality, research guidance, career guidance, and career support are included, the independent role of contact frequency is no longer consistently significant. By contrast, supervisory relationship quality, research guidance, career guidance, and career support remain positively and robustly associated with overall PhD satisfaction across different model specifications. These findings suggest that PhD students’ learning experience is more consistently associated with the quality of supervisor support than with contact frequency itself.

This study makes three main contributions. First, at the level of research question, it distinguishes supervisory contact frequency from the quality of supervisor support, thereby addressing whether increasing supervisor–student contact is sufficient to improve doctoral learning experience. Second, in terms of variable construction, it disaggregates supervisor support into multiple dimensions, including supervisory relationship, research guidance, career guidance, and career support, rather than treating supervisor support as a single undifferentiated construct. Third, empirically, it uses a global publicly available PhD survey dataset to conduct systematic model-based analysis, providing new evidence for understanding the relationship between supervisor support and doctoral learning experience across diverse doctoral training contexts.

Accordingly, the study addresses three research questions. **RQ1:** How is supervisory contact frequency associated with overall PhD satisfaction? **RQ2:** How are supervisory relationship quality, research guidance, career guidance, and supervisor career support associated with overall PhD satisfaction? **RQ3:** Does supervisory contact frequency retain an independent association with overall PhD satisfaction after support-quality dimensions are taken into account, and do the observed patterns remain stable across alternative model specifications and outcome measures?

## Literature review

PhD students’ learning experience is commonly understood as their overall experience of research training, academic interaction, institutional support, and career development. It is often reflected in indicators such as satisfaction, research engagement, mental health, academic persistence, and career expectations. Recent studies suggest that the doctoral experience is not determined solely by individual ability, but is embedded in multiple conditions, including supervisory relationships, research communities, peer support, departmental environment, and life pressures. Dericks et al. [1] found that the quality of supervisors, departments, and peers all affected PhD student satisfaction, with supervisor supportiveness playing a particularly important role. Corcelles-Seuba et al. [5], Rönkkönen et al. [6], and Cao et al. [7] further showed that supervisor support and research community support contribute to PhD students’ research engagement and academic involvement. Negative doctoral experiences are also frequently associated with psychological stress, burnout, dropout intentions, and the risk of interrupting doctoral studies [8–10].

Supervisor support is not a single behaviour, but a combination of relational, academic, and developmental support. Relational support involves trust, respect, communication, and emotional availability; research guidance concerns topic selection, methodology, dissertation feedback, and academic norms; career support involves career discussions, understanding of non-academic pathways, and access to development opportunities. Wang et al. [2] distinguished the roles of high-quality supervision and peer support in shaping PhD students’ research experience, while emphasizing that supervisor support remains a core resource in doctoral research training. From a multicultural perspective, Guarimata-Salinas et al. [11] argued that contemporary doctoral supervisors are no longer merely dissertation managers, but increasingly perform educational, relational, and developmental functions. Mavrogalou-Foti et al. [12] also found a significant association between supervisory relationships and doctoral students’ mental health outcomes. Taken together, these studies suggest that supervisor support should be disaggregated into specific dimensions rather than treated as a broad and undifferentiated construct [3,13].

Existing studies often regard supervisory contact frequency as an important condition in doctoral training, yet contact frequency does not necessarily equate to effective supervision. Cornér et al. [8] examined the relationships among supervision frequency, supervision satisfaction, and burnout. Anttila et al. [13] further compared supervisors’ and supervisees’ perceptions of supervisory support and supervision frequency, showing that the amount of contact and the extent to which students receive the support they need are not always aligned. Tikkanen et al. [3] incorporated both supervision quantity and supervision quality into their analysis of PhD candidates’ and supervisors’ well-being, suggesting that frequent interaction is more likely to improve the doctoral experience only when it is appropriate and supportive. González-Betancor and Dorta-González [9] found that insufficient contact with supervisors was associated with the risk of interrupting doctoral studies, although mental health remained a stronger risk factor. In this sense, contact frequency may be better understood as a formal condition under which support can occur, whereas support quality is closer to the substantive mechanism through which doctoral learning experiences are shaped.

The career aims of doctoral education are also shifting from preparation for a single academic career path toward more diverse forms of career development. Carriero et al. [14] and Boman et al. [15] showed that supervisors, funding, and collaborative experiences during doctoral training can influence the career pathways of doctorate holders. Chen et al. [16], in a review of doctoral employability in Australia, argued that doctoral education needs to respond more systematically to cross-sector employment and the development of transferable skills. Skakni et al. [17], in a scoping review of non-academic careers among PhD graduates, similarly showed that careers beyond academia have become an important issue in doctoral development research. Rönkkönen et al. [18] further demonstrated that the transition of PhD holders into non-academic work is also shaped by social support. Whether supervisors can openly discuss non-academic careers, provide career advice, and support career training has therefore become an important component of how PhD students evaluate their training experience.

Overall, existing research has consistently shown that PhD students’ learning experience is shaped not merely by individual ability or research outputs, but by supervisory relationships, research communities, peer support, departmental environments, and career prospects. Among these factors, supervisor support has repeatedly been identified as an important influence on PhD student satisfaction, research engagement, mental health, and academic persistence. Recent studies have also begun to move beyond a general notion of “supervision”, emphasizing that supervisors shape students’ overall evaluation of doctoral training not only through methodological guidance, dissertation feedback, and academic norms, but also through communication, trust, career advice, and emotional support.

A remaining limitation in the literature is that the internal structure of supervisor support has not been sufficiently differentiated, especially in terms of the relative roles of supervisory contact frequency and support quality. Longer contact time with supervisors does not necessarily mean that PhD students receive more effective support; what matters is whether contact contains high-quality relational support, research guidance, and career support. Responding to this gap, the present study distinguishes supervisor support into two levels: supervisory contact frequency and the quality of supervisor support. It further examines how supervisory relationship quality, research guidance, career guidance, and career support are associated with overall PhD satisfaction and related outcomes of the doctoral learning experience.

## Materials and methods

### Data source and ethics

This study used publicly available, anonymized data from Nature’s Graduate Survey 2025 for secondary analysis [4]. The survey was conducted in May and June 2025 and received 3,785 self-selecting responses from PhD students across 107 countries. The authors did not recruit or interact with participants, had no access to directly identifiable personal information, and analyzed only publicly available, de-identified records. The Graduate School of Shaanxi Normal University reviewed the study protocol and determined that the present secondary analysis qualified for exemption from further institutional ethical review. Because no new participants were recruited and no additional data were collected, additional written or verbal informed consent was waived. No attempt was made to re-identify any participant.

The dataset is well suited to examining the relationship between PhD students’ learning experience and supervisor support. First, the survey includes measures of overall PhD satisfaction, supervisory contact frequency, supervisory relationship quality, research guidance, career guidance, and career support, allowing this study to distinguish different dimensions of supervisor support. Second, the sample has broad international coverage, making it possible to examine the association between supervisor support and PhD students’ learning experience across diverse national and doctoral training contexts.

### Variables and measures

The dependent variable is overall PhD satisfaction, which captures PhD students’ overall evaluation of their doctoral experience. The key explanatory variables consist of two dimensions: supervisory contact frequency and the quality of supervisor support. Supervisory contact frequency is divided into three categories: less than 1 hour per week, 1–3 hours per week, and more than 3 hours per week. The category “less than 1 hour per week” is used as the reference group.

The quality of supervisor support includes supervisory relationship quality, research guidance, career guidance, and the supervisor career support index. The supervisor career support index is constructed as the mean of four items from Q20 that capture openness to non-academic careers, encouragement of career training and events, time for frank career conversations, and useful advice for careers outside academia. To facilitate replication, Table 1 reports the relevant Nature Careers 2025 survey question codes and item content together with the coding and analytical treatment used in the present study. To examine the overall explanatory role of supervisor support, this study also constructs an overall supervisor support index by standardizing supervisory relationship quality, research guidance, career guidance, and the supervisor career support index, and then taking their mean.

**Table 1:** Variable definitions, source survey items, and analytical coding.

Analytical variable	Nature Careers 2025 survey question / item	Measurement, coding, and analytical use
Overall PhD satisfaction (primary outcome)	Q11 – overall satisfaction with the PhD experience.	Used as the main ordered outcome; higher scores indicate greater satisfaction.
Supervisory contact frequency (key explanatory variable)	Q18 – weekly one-on-one contact time with the supervisor.	Recoded into less than 1 hour/week (reference), 1–3 hours/week, and more than 3 hours/week.
Supervisory relationship quality (key explanatory variable)	Q14 – multi-item satisfaction battery; selected item on the relationship with the supervisor.	Higher values indicate greater satisfaction with the supervisory relationship; entered as a support-quality predictor.
Research guidance (key explanatory variable)	Q14 – selected item on research-related guidance within the PhD-experience satisfaction battery.	Higher values indicate greater satisfaction with research guidance; entered as a support-quality predictor.
Career guidance (key explanatory variable)	Q14 – selected item on career-pathway guidance/advice within the PhD-experience satisfaction battery.	Higher values indicate greater satisfaction with career guidance; entered as a support-quality predictor.
Supervisor career support index (key explanatory variable)	Q20 – supervisor career-support battery covering openness to non-academic careers, encouragement of career training/events, time for frank career conversations, and useful advice for careers outside academia.	Mean of the four Q20 items; higher values indicate stronger supervisor career support.
Overall supervisor support index (composite explanatory variable)	Derived from supervisory relationship quality, research guidance, career guidance, and the supervisor career support index.	Each component is standardized and the standardized components are averaged to form the composite index.
Individual and doctoral background controls	Q3a (doctoral stage), Q3b (study mode), Q17 (weekly PhD working hours), plus survey mobility and demographic items.	Controls include doctoral stage, study mode, weekly working hours, study outside the home country, age group, gender, minority ethnic identity, and care responsibilities.
External pressure factors (extended controls)	Q10 (cost-of-living items), Q15 (current PhD concerns, including financial pressure), and Q24 (discrimination/harassment experiences).	Added in extended-control models to test whether the estimated associations for supervisor-support variables remain stable after external pressures are taken into account.
Initial expectations (alternative outcome)	Q12 – item assessing whether the reality of the PhD experience aligns with initial expectations.	Used as an alternative outcome in robustness analysis; higher values indicate closer alignment with initial expectations.

Note: Nature Careers 2025 survey question codes are reported to make the construction of the analytical variables traceable to the source survey. For multi-item questions (notably Q14 and Q20), the relevant item content is summarized. Derived indices and recoding decisions are described in the third column. See the Nature’s Graduate Survey 2025 data source [4].

The control variables include doctoral stage, study mode, weekly working hours on the PhD, study outside the home country, age group, gender, minority ethnic identity, and care responsibilities. In the robustness checks, this study further includes cost-of-living concerns, financial pressure, and experience of discrimination or harassment to examine the stability of the supervisor support variables. Table 1 provides the source-survey mapping and operationalization for the outcome, explanatory, control, and extended variables.

### Statistical analysis

This study first uses ordinary least squares (OLS) regression as the baseline model to examine the association between supervisory contact frequency, the quality of supervisor support, and overall PhD satisfaction. The baseline model is specified as follows:


$$\begin{array}{c} Satisfaction_{i}=\alpha +\beta_{\text{1}}Contact_{i}+\beta_{\text{2}}Support_{i}+\gamma X_{i}+\varepsilon_{i} \end{array}$$

Where Satisfactioni denotes the overall PhD satisfaction of respondent i; Contacti denotes supervisory contact frequency; Supporti denotes the quality of supervisor support, including supervisory relationship quality, research guidance, career guidance, and the supervisor career support index; Xi denotes the vector of control variables; and εi is the error term.

Because overall PhD satisfaction is measured as an ordered response, this study further uses an ordered logit model as a robustness check. The model is specified as follows:


$$\begin{array}{c} Pr(Y_{i}\leq j)=\Lambda (\tau_{j}-\beta_{\text{1}}Contact_{i}-\beta_{\text{2}}Support_{i}-\gamma X_{i}) \end{array}$$

Where j denotes the threshold of the satisfaction category, τj is the corresponding cut point, and Λ denotes the logistic cumulative distribution function. To further examine the stability of the results, this study also conducts supplementary analyses using a binary logit model for high satisfaction, an alternative outcome model, and models with extended control variables.

## Results

### Descriptive statistics

The descriptive statistics show that the mean value of overall PhD satisfaction is 4.946, indicating a moderate-to-high level of satisfaction in the sample. The mean values of supervisory relationship quality and research guidance are 5.252 and 4.821, respectively, which are higher than those of career guidance and the supervisor career support index. This suggests that, compared with academic training support, PhD students receive relatively weaker support for career development. Supervisory contact frequency is concentrated mainly in the categories of less than 1 hour per week and 1–3 hours per week, while the proportion of students with more than 3 hours of weekly supervisory contact is relatively small. The sample also shows heterogeneity in doctoral stage, study mode, international mobility background, gender, minority ethnic identity, and care responsibilities, providing a basis for controlling individual and doctoral background differences in the multivariate models. Table 2 reports the descriptive statistics for the main analytical variables.

**Table 2:** Descriptive statistics of the main variables.

Variable	N/Valid N	Mean / Percentage	SD / Count
Overall PhD satisfaction	3785	4.946	1.389
Supervisory relationship quality	3785	5.252	1.932
Research guidance	3785	4.821	1.936
Career guidance	3785	4.397	1.939
Supervisor career support index	3785	3.483	1.120
Less than 1 hour per week	3631	50.73%	1842
1–3 hours per week	3631	37.62%	1366
More than 3 hours per week	3631	11.65%	423
Within first two years	3785	37.91%	1435
Years 3–5	3785	53.55%	2027
Year 6 or above	3785	8.53%	323
Full-time	3750	87.84%	3294
Part-time	3750	12.16%	456
Study outside home country	3785	32.55%	1232
Female	3785	43.54%	1648
Minority ethnic identity	3785	24.51%	929
Care responsibilities	3694	31.05%	1147

Note: Continuous variables are reported as means and standard deviations; categorical variables are reported as percentages and counts. “Less than 1 hour per week” is the reference category for supervisory contact frequency in the regression models. Age group and weekly working hours on PhD are included as controls but are not reported here because their validated category-level distributions have not been provided.

### Baseline OLS results: Relative roles of supervisory contact frequency and support quality

Table 3 reports the OLS estimates of the association between supervisor support and overall PhD satisfaction. To compare the relative explanatory roles of supervisory contact frequency and the quality of supervisor support, this study estimates a series of specifications. Column (1) includes supervisory contact frequency, with the category “less than 1 hour per week” as the reference group. Column (2) further adds supervisory relationship quality, research guidance, and career guidance. Column (3) additionally includes the supervisor career support index. Column (4) replaces the separate support-quality indicators with the overall supervisor support index.

**Table 3:** OLS models predicting overall PhD satisfaction.

Variables	(1)	(2)	(3)	(4)
1–3 hours per week	0.484^***^	0.026	0.015	
	(0.048)	(0.043)	(0.043)	
More than 3 hours per week	0.669^***^	0.108	0.093	
	(0.073)	(0.069)	(0.069)	
Supervisory relationship quality		0.161^***^	0.145^***^	
		(0.017)	(0.017)	
Research guidance		0.157^***^	0.153^***^	
		(0.018)	(0.017)	
Career guidance		0.125^***^	0.114^***^	
		(0.015)	(0.016)	
Supervisor career support index			0.083^***^	
			(0.024)	
Overall supervisor support index				0.913^***^
				(0.027)
Controls	Yes	Yes	Yes	Yes
N	3,515	3,515	3,515	3,661
R²	0.087	0.333	0.336	0.331

Note: Standard errors are reported in parentheses. The reference category for supervisory contact frequency is “less than 1 hour per week.” All models control for doctoral stage, study mode, weekly working hours on PhD, study outside home country, age group, gender, minority ethnic identity, and care responsibilities. * p < .10, ** p < .05, *** p < .01.

Column (1) focuses on the association between supervisory contact frequency and overall PhD satisfaction. Compared with PhD students who had less than 1 hour of weekly contact with their supervisor, those who had 1–3 hours of weekly contact reported significantly higher overall satisfaction, with a coefficient of 0.484. The coefficient for more than 3 hours of weekly contact is 0.669 and is also significant at the 1% level. These results indicate that, before accounting for the quality of supervisor support, supervisory contact frequency is positively associated with overall PhD satisfaction. In simpler specifications, more frequent contact appears to explain part of the variation in satisfaction.

The pattern changes substantially once the quality of supervisor support is included. Column (2) shows that, after supervisory relationship quality, research guidance, and career guidance are added, the coefficients for 1–3 hours and more than 3 hours of weekly supervisory contact decline to 0.026 and 0.108, respectively, and both lose statistical significance. By contrast, supervisory relationship quality, research guidance, and career guidance are all positive and significant at the 1% level, with coefficients of 0.161, 0.157, and 0.125, respectively. The R² also increases markedly to 0.333. This result suggests that the significant association between contact frequency and satisfaction in the simpler specification may largely reflect the relational, research-related, and career-related support embedded in supervisory interactions, rather than the independent effect of contact time itself.

Column (3) further includes the supervisor career support index, and the results remain stable. The coefficient of the supervisor career support index is 0.083 and is significant at the 1% level. Supervisory relationship quality, research guidance, and career guidance also remain positively and significantly associated with overall PhD satisfaction, while supervisory contact frequency remains statistically insignificant. This finding has two implications. First, overall PhD satisfaction is associated not only with research-related supervision but also with whether supervisors respond to students’ career development needs. Second, career support does not replace the role of relational support or research guidance; rather, it constitutes an additional dimension of supervisor support that is independently associated with PhD student satisfaction.

Column (4) uses the overall supervisor support index instead of the separate support-quality indicators. The coefficient of the overall supervisor support index is 0.913 and is significant at the 1% level, with an R² of 0.331. This result shows that when supervisory relationship quality, research guidance, career guidance, and career support are integrated into a composite measure of supervisor support, the overall support index remains strongly and positively associated with overall PhD satisfaction. Compared with the contact-frequency specification in Column (1), the overall supervisor support index has substantially greater explanatory power.

Taken together, Table 3 reveals a clear empirical pattern: supervisory contact frequency is significantly associated with overall PhD satisfaction in a simpler specification, but this association is no longer robust once the quality of supervisor support is included. By contrast, supervisory relationship quality, research guidance, career guidance, and career support remain consistently and positively associated with satisfaction across specifications. This finding suggests that PhD students’ learning experience is more consistently associated with the quality of supervisor support than with supervisory contact frequency itself. Contact time may be understood as a formal condition under which support can occur, but whether it translates into a positive doctoral experience depends on whether the interaction contains effective relational, research-related, and career-development support.

**Fig 1:**
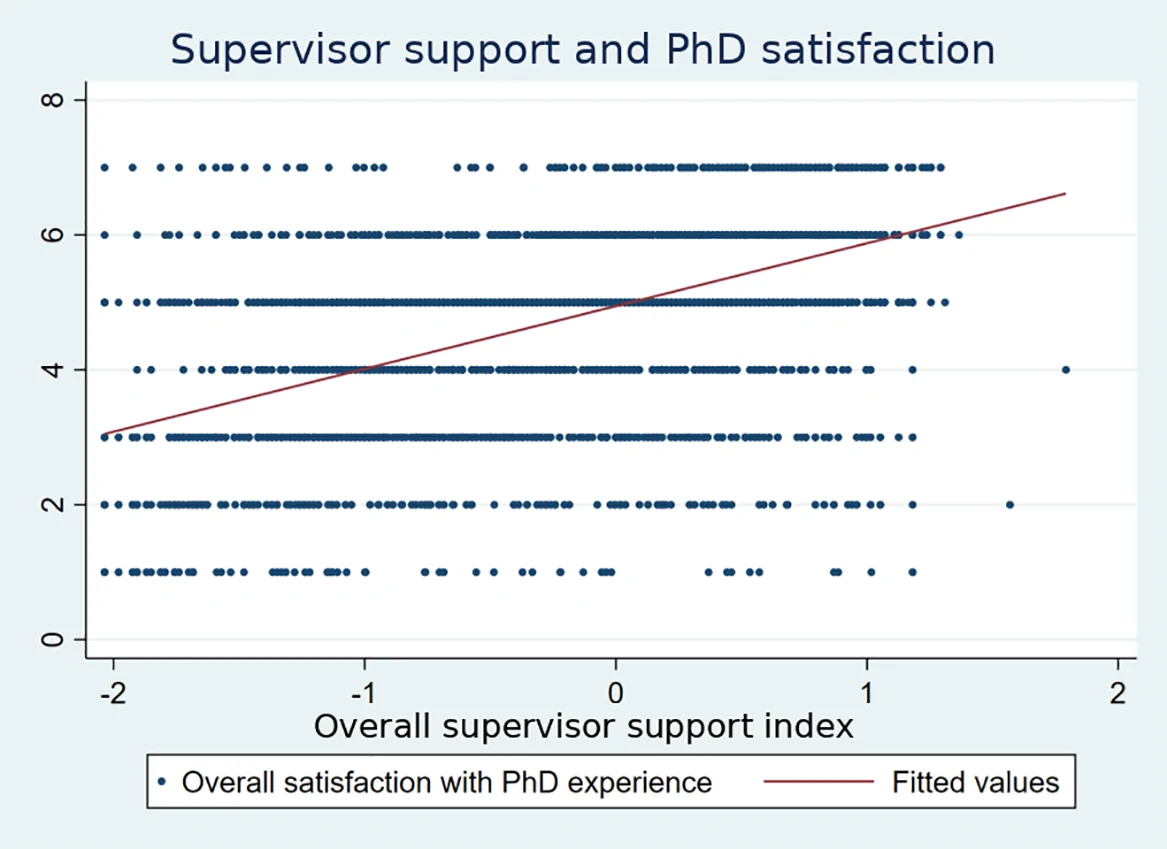
Relationship between the overall supervisor support index and overall PhD satisfaction.

Fig 1 further illustrates the relationship between the overall supervisor support index and overall PhD satisfaction. The positive visual trend is consistent with the fully adjusted OLS estimate in Table 3, Column (4), where the overall supervisor support index has a coefficient of 0.913 (SE = 0.027, p < .01; R² = 0.331). By comparison, the contact-frequency coefficients lose statistical significance after support-quality dimensions are introduced in Columns (2) and (3). Taken together, the visual and regression evidence indicates that variation in PhD satisfaction is more consistently aligned with the quality and developmental content of supervisor support than with contact time alone.

### Ordered logit and marginal effects

Because overall PhD satisfaction is measured as an ordered response, relying solely on OLS may not fully account for the measurement properties of the dependent variable. To examine whether the baseline findings depend on model specification, this study further estimates an ordered logit model. Table 4 reports the main results.

**Table 4:** Ordered logit model predicting overall PhD satisfaction.

Variables	Coefficient
1–3 hours per week	0.022 (0.070)
More than 3 hours per week	0.207^*^ (0.121)
Supervisory relationship quality	0.239^***^ (0.029)
Research guidance	0.268^***^ (0.028)
Career guidance	0.221^***^ (0.027)
Supervisor career support index	0.138^***^ (0.039)
Controls	Yes
N	3,515

Note: Standard errors are reported in parentheses. The reference category for supervisory contact frequency is “less than 1 hour per week.” The control variables are the same as those in Table 3. Cut points are omitted for brevity. * p < .10, ** p < .05, *** p < .01.

The results show that the support-quality variables remain positively and significantly associated with higher levels of overall PhD satisfaction. The coefficients of supervisory relationship quality, research guidance, career guidance, and the supervisor career support index are 0.239, 0.268, 0.221, and 0.138, respectively, and all are significant at the 1% level. This indicates that, when overall PhD satisfaction is treated as an ordered outcome, higher-quality supervisor support is still associated with a greater likelihood of being in a higher satisfaction category. By contrast, the independent role of supervisory contact frequency remains weak. The coefficient for 1–3 hours per week is 0.022 and is not statistically significant; the coefficient for more than 3 hours per week is 0.207 and is only marginally significant at the 10% level.

This pattern is consistent with the OLS results. After the quality of supervisor support is controlled for, supervisory contact frequency does not show a stable independent association with satisfaction, whereas supervisory relationship quality, research guidance, career guidance, and career support remain significant across model specifications. This further suggests that differences in satisfaction levels are not primarily explained by contact time itself, but by the quality of support embedded in supervisory interactions.

The marginal effects provide additional evidence on the substantive meaning of these associations. Holding other variables constant, a one-unit increase in supervisory relationship quality is associated with an increase of approximately 1.69 percentage points in the probability of being in the highest satisfaction category. A one-unit increase in research guidance is associated with an increase of approximately 1.89 percentage points. The corresponding increases for career guidance and the supervisor career support index are approximately 1.56 and 0.97 percentage points, respectively. At the same time, these support-quality variables reduce the probability of being in lower satisfaction categories. These results indicate that supervisor support quality is not only statistically significant but also associated with consistent shifts in the distribution of satisfaction levels.

### Robustness checks

To further examine the stability of the main findings, this study conducts three robustness checks. First, overall PhD satisfaction is converted into a binary indicator of high satisfaction, and a binary logit model is estimated. Second, whether the PhD experience met students’ initial expectations is used as an alternative outcome to examine whether supervisor support is also associated with broader evaluations of the doctoral experience. Third, cost-of-living concerns, financial pressure, and experience of discrimination or harassment are added to the baseline model to test whether the role of supervisor support quality remains stable after accounting for external pressure factors.

Table 5 shows that, in the binary logit model predicting high satisfaction, supervisory relationship quality, research guidance, career guidance, and the supervisor career support index remain positive and statistically significant, with coefficients of 0.231, 0.227, 0.179, and 0.127, respectively. The two supervisory contact frequency categories are not statistically significant. This result indicates that even when satisfaction is recoded as a binary outcome, the quality of supervisor support remains significantly associated with the likelihood of being in the high-satisfaction group, whereas contact frequency still does not show a stable explanatory role.

**Table 5:** Robustness checks.

Variables	High satisfaction	Initial expectations	Extended controls
1–3 hours per week	0.014 (0.105)	−0.038 (0.071)	0.015 (0.043)
More than 3 hours per week	0.105 (0.180)	0.027 (0.113)	0.101 (0.068)
Supervisory relationship quality	0.231^***^ (0.032)	0.193^***^ (0.028)	0.143^***^ (0.017)
Research guidance	0.227^***^ (0.034)	0.232^***^ (0.027)	0.154^***^(0.018)
Career guidance	0.179^***^ (0.033)	0.175^***^ (0.026)	0.106^***^ (0.016)
Supervisor career support index	0.127^**^ (0.051)	0.124^***^ (0.039)	0.079^***^ (0.024)
Cost-of-living concerns			−0.075^***^ (0.020)
Financial pressure			−0.145^***^ (0.040)
Discrimination or harassment experience			−0.159^***^ (0.040)
Controls	Yes	Yes	Yes
N	3,515	3,515	3,515

Note: The first column reports a binary logit model predicting high satisfaction. The second column uses whether the PhD experience met initial expectations as an alternative outcome. The third column reports the OLS model with extended controls. Standard errors are reported in parentheses. All models control for doctoral stage, study mode, weekly working hours on PhD, study outside home country, age group, gender, minority ethnic identity, and care responsibilities. * p < .10, ** p < .05, *** p < .01.

The alternative outcome model further supports this conclusion. When the outcome is replaced by whether the PhD experience met students’ initial expectations, supervisory relationship quality, research guidance, career guidance, and the supervisor career support index are all positive and significant at the 1% level, while supervisory contact frequency remains insignificant. This finding suggests that supervisor support quality is associated not only with students’ satisfaction with their current doctoral experience, but also with their broader evaluation of whether the PhD experience corresponds to their initial expectations. In other words, supervisor support quality is linked not merely to a single satisfaction indicator, but to students’ overall assessment of doctoral training and development opportunities.

The extended-control model further includes cost-of-living concerns, financial pressure, and experience of discrimination or harassment. The results show that all three external pressure factors are significantly and negatively associated with overall PhD satisfaction. Nevertheless, the supervisor support quality variables remain positive and statistically significant. The coefficients of supervisory relationship quality, research guidance, career guidance, and the supervisor career support index are 0.143, 0.154, 0.106, and 0.079, respectively. This indicates that although PhD satisfaction is indeed shaped by external pressures, the association between supervisor support quality and overall satisfaction does not disappear after these factors are taken into account.

## Discussion

### Principal findings and interpretation

Overall, the results consistently show that supervisory contact frequency is positively associated with overall PhD satisfaction in reduced specifications, but this association is no longer stable after the quality of supervisor support is included. By contrast, supervisory relationship quality, research guidance, career guidance, and career support remain positively associated with satisfaction across OLS, ordered logit, binary logit, alternative-outcome, and extended-control models. The central empirical finding is therefore not that “more contact is always better,” but that contact time alone is insufficient to explain PhD students’ learning experience; what matters more consistently is whether supervisory interaction contains high-quality relational, research-related, and career-development support. Supervisory contact frequency captures the amount of interaction, but it does not reveal whether the content of that interaction is effective. Even when PhD students maintain frequent contact with their supervisors, interactions dominated by administrative communication, progress pressure, or formalized feedback may make only a limited contribution to the learning experience. By contrast, supervisory relationship quality, research guidance, career guidance, and career support more directly capture the support resources that students receive during doctoral training, including whether they feel respected and understood, receive clear research feedback, can discuss career development, and are supported in exploring diverse career options. These dimensions are therefore closer to the substantive foundations of PhD student satisfaction.

The results also suggest that PhD students’ learning experience is multidimensional. Supervisory relationship quality provides a foundation of trust and communication safety; research guidance helps students navigate uncertainty in the research process and advance their academic work; career guidance and career support respond to the shift in doctoral education from preparation for a single academic career path toward more diverse forms of career development. PhD students’ evaluation of their doctoral experience therefore depends not only on dissertation progress, but also on supervisory relationships, development opportunities, and future career possibilities. From the perspective of doctoral education quality assurance, measuring supervision quality solely by meeting frequency is insufficient. Meeting frequency may serve as a minimum process requirement, but it should not be equated with effective supervision. More meaningful indicators should focus on the content and quality of supervisory interaction, including whether feedback is specific, whether the relationship is supportive, whether research guidance is clear, whether career discussions are open, and whether supervisors support students in exploring broader development pathways. Improving the quality of doctoral education therefore requires not simply increasing the number of meetings, but strengthening the substantive quality of supervisor support.

Drawing on the publicly available anonymized data from the Nature Careers 2025 Global PhD Survey, this study empirically examined the relationship between supervisor support and PhD students’ learning experience. The findings show that supervisory contact frequency is positively associated with overall PhD satisfaction at the descriptive level and in simpler regression models. In other words, PhD students who have more weekly contact with their supervisors tend to report higher levels of satisfaction. However, after supervisory relationship quality, research guidance, career guidance, and supervisor career support are included, the independent explanatory role of supervisory contact frequency becomes substantially weaker and is no longer consistently significant. By contrast, supervisory relationship quality, research guidance, career guidance, and supervisor career support remain positively and robustly associated with overall PhD satisfaction across different model specifications. The ordered logit model, marginal effects, binary logit model for high satisfaction, alternative outcome model, and extended-control models all support this conclusion. These findings suggest that PhD students’ learning experience is more consistently associated with the quality of supervisor support than with contact frequency itself. Contact time may provide opportunities for interaction, but what enters students’ evaluation of doctoral satisfaction is the relational, research-related, and career-development support embedded in supervisory interaction.

This finding has theoretical implications for doctoral education research. Existing studies have widely emphasized the importance of supervisor support for doctoral training quality. However, if supervisor support is treated only as an overall construct, its internal structural differences may be obscured. This study distinguishes supervisory contact frequency from the quality of supervisor support, and further disaggregates support quality into supervisory relationship quality, research guidance, career guidance, and career support. In doing so, it shows that PhD students’ evaluations of their learning experience are not shaped simply by whether supervisors are present, but by the specific content and mode of supervisory interaction. Career guidance and career support are particularly noteworthy because they show independent explanatory roles in the models. This indicates that the responsibilities of doctoral supervisors in contemporary doctoral education have extended beyond dissertation supervision and academic training in the traditional sense, increasingly encompassing career development, understanding of diverse career pathways, and support for non-academic career options. PhD students’ judgments of the doctoral experience therefore include not only their evaluation of research progress, but also their broader assessment of development opportunities, future career possibilities, and the quality of supervisory relationships.

### Implications for doctoral training

Based on these findings, doctoral training quality assessment should not rely excessively on formal indicators such as meeting frequency. Supervisory contact frequency may serve as a basic process requirement to prevent prolonged absence of supervision, lack of guidance, or delayed feedback, but it should not be equated with high-quality supervision. Universities and graduate training units should pay greater attention to the substantive content of supervisory interaction and incorporate supervisory relationship quality, feedback quality, career guidance quality, and developmental support into the assessment of doctoral training processes. Rather than merely specifying how often supervisors and PhD students should meet each week or month, it is more important to establish evaluation mechanisms that can identify effective supervision. Such mechanisms should consider whether PhD students receive clear research feedback, whether they can communicate with supervisors in an open and trustworthy manner, whether they receive concrete support when research difficulties arise, and whether they can discuss academic and non-academic career development meaningfully. Only when attention shifts from interaction frequency to interaction quality can supervisor support be translated into a positive doctoral learning experience.

In terms of supervisor development, universities need to expand supervisor training beyond research capacity and dissertation management to include relational support, feedback competence, and career development guidance. Doctoral supervisors certainly need strong academic expertise, but PhD student satisfaction does not depend solely on supervisors’ academic reputation or research productivity. The results of this study suggest that students more directly experience supervisors’ availability, feedback effectiveness, communication practices, and developmental support during the training process. Universities can help supervisors improve their supervisory practices through supervisor training, sharing of supervisory experience, assessment of supervision cases, and systematic PhD student feedback mechanisms. Career support deserves particular attention. Supervisors should be encouraged to discuss diverse post-PhD career pathways more openly and avoid treating non-academic career choices as a form of academic failure. For PhD students, opportunities to discuss career directions in universities, research institutions, industry, government, and social organizations can help them develop more realistic and stable career expectations.

At the institutional level, doctoral training needs to move from a dissertation-completion orientation toward a whole-process developmental support orientation. The doctoral dissertation remains a core outcome of doctoral education, but students’ learning experience and developmental quality are not determined by dissertation output alone. Training units can establish more differentiated support systems by integrating research progress assessment, psychological support, career development services, and supervision quality evaluation. In areas where career development support is relatively weak, universities can provide supplementary resources through career development courses, cross-sector internship opportunities, alumni career networks, industry mentor schemes, and non-academic career counselling. Supervisors may not be able to provide all career resources independently, but their attitudes toward diverse career pathways and their modes of support can influence whether PhD students are willing to seek such resources. Improving the quality of doctoral education requires the joint participation of supervisors, departments, graduate schools, and career development services, rather than placing all support responsibilities solely on individual supervisors.

### Limitations and future research

This study has several limitations. First, it uses cross-sectional survey data from a self-selecting sample. The estimates therefore identify associations between supervisor support and PhD students’ learning experience, not causal effects, and the observed relationships should not be interpreted as evidence that changes in supervisor support necessarily produce changes in satisfaction. Second, the main variables are based on students’ self-reported evaluations, which may be influenced by subjective perceptions, individual expectations, and cultural background differences. Third, although the dataset covers 107 countries and has broad international scope, this study does not further examine heterogeneity across countries, disciplines, and doctoral training systems. Future research could use longitudinal designs to examine within-student changes over time, combine survey evidence with qualitative interviews to clarify how specific supervisory practices are experienced, and estimate country- or discipline-specific models to test whether the observed associations vary across doctoral training contexts. Future studies could also examine interactions among supervisor support, departmental environment, peer support, and career-development resources to provide a more comprehensive account of the support structure in doctoral training.

## Conclusions

Across a global sample of PhD students, the quality of supervisory relationships, research guidance, career guidance, and career support was more consistently associated with doctoral satisfaction than contact frequency alone. Meeting frequency may create opportunities for support, but it should not be treated as a sufficient indicator of supervision quality. Doctoral programmes should therefore evaluate and develop supervision in terms of the substantive relational, research-related, and career-development support that students receive.
